# Estimating the Effect of the Kappa Casein Genotype on Milk Coagulation Properties in Israeli Holstein Cows

**DOI:** 10.3390/ani14010054

**Published:** 2023-12-22

**Authors:** Yaniv Lavon, Joel I. Weller, Yoel Zeron, Ephraim Ezra

**Affiliations:** 1 Israel Cattle Breeders Association, Caesaria Industrial Park, Caesarea 38900, Israel; joel.weller@mail.huji.ac.il (J.I.W.);; 2 Sion Artificial Insemination Center, Gadara 7057102, Israel; yoel@sion-israel.com

**Keywords:** kappa casein, coagulation, genotype, dairy cow

## Abstract

**Simple Summary:**

Milk with favorable coagulation properties requires a shorter coagulation time and yields a higher curd firmness. Milk from cows with the B allele of kappa casein has an advantage in terms of the quantity of cheese produced compared to milk from cows with the A or E alleles. We show a clear advantage of genotypes that include the B allele over those without it. In addition, the coagulation properties in primiparous cows were favorable compared to later parity cows. The proportion of the B allele in the population can be increased by insemination of cows using genotype AB and BB bulls.

**Abstract:**

In Israel, about 26% of produced milk is used to produce hard cheeses and 29% for soft cheeses. Milk with preferred coagulation properties requires a shorter coagulation time and yields a higher curd firmness than milk with inferior coagulation properties. Studies have shown that milk from cows with the B allele of kappa casein (κ-CN) produces more cheese than milk from those with A and E alleles. There is evidence that milk from AE or EE genotype cows is unsuitable for cheese production. In the early 1990s, the proportion of the B allele in Israeli Holstein cattle was about 17%, similar to its prevalence in the Holstein population worldwide. In recent years, however, its proportion has increased to about 40%. We analyzed milk coagulation properties as a function of the cow’s κ-CN genotype, including time in minutes until the beginning of coagulation and curd firmness after 60 min—measured in volts via an optigraph device and scored on a scale of 0–4 by a laboratory technician. Cow selection was based on their sire’s genotype, so that there would be sufficient genotypes that include the rare E allele. A total of 359 cows were sampled from 15 farms: 64 with genotype AA, 142 with AB, 41 with AE, 65 with BB, and 47 with BE. Data were analyzed via the general linear model procedure of SAS. We found the following: (a) There were significant differences between genotypes for optigraph-measured curd firmness. In a multi-comparison test, the BB genotype gave the highest curd firmness, and AB and BE showed a significant advantage compared to AA and AE (9.4, 8.6, 8.4, 6.9, 6.8 V, respectively). Assuming a frequency of about 55% for the A allele, about 30% of the milk delivered to dairy plants comes from AA cows. (b) There was a significant difference between the genotypes in technician-observed curd firmness, with BB scoring significantly higher than AA and AE. (c) The optigraph-measured curd firmness was significantly higher for milk from primiparous cows as compared to milk from second, third, or fourth lactation cows (8.9, 7.8, 7.9, 7.7 V, respectively). The technician-observed curd firmness was significantly higher for primiparous vs. multiparous cows. There was a clear advantage in curd firmness for genotypes that included the B allele compared to those with AA and AE genotypes. We can increase the proportion of the B allele in the population by insemination of cows using bulls with the genotypes AB and BB. This factor should therefore be included in the selection index.

## 1. Introduction

Milk’s coagulation ability is important for cheese production. Milk coagulation and curd-firming processes have been widely studied in recent decades, and milk protein fractions have been identified as the principal factors in these processes [1]. In most milk-producing countries, a large and growing fraction of the produced milk is used to make cheese [2,3,4]. In Israel, around 26% of produced milk is used to produce hard cheeses and 29% for soft (white) cheeses [5]. The milk’s ability to coagulate (time and quality) is economically significant; milk with preferential coagulation properties will yield larger amounts of cheese with the desired content than milk with inferior coagulation properties [2]. Milk coagulation properties are influenced by breed [1], somatic cell count (SCC) and bacteriology [6], milk protein composition and casein composition [7,8,9], and stage of lactation [10], among other factors. In addition, cheese-making traits can be affected by environmental factors such as feeding, udder health, season, and physiological stage (e.g., parity, lactation stage), but they are also genetically influenced [4,11,12]. Milk coagulation properties are heritable according to Ikonen et al. [13] and can therefore be improved by selective breeding. In dairy products, the kappa casein (κ-CN) component in milk proteins is responsible for coagulation. κ-CN exists as variants AA, AB, BB, AC, BC, and AE in bovine milk, with allele C being more common in Jersey cows and not present in Holstein cows.

In the Holstein population, the gene encoding κ-CN has three allelic variants: A, B, and E. Most studies show an advantage for the B allele in protein and CN contents. Many studies have confirmed that milk containing the BB variant of κ-CN has a faster and firmer gelling ability and is more suitable for cheese production than other variants [14,15,16]. According to Ng-Kwai-Hang [17], milk with the BB variant of κ-CN shows a reduced coagulation time (by 10–40%) and increased curd firmness (by 20–140%) compared to milk with an AA variant. In particular, the most consistent effect was found for CSN3 (κ-CN) variant B, which has been shown to have a positive effect on κ-CN concentration in milk [18,19,20], and to be associated with a smaller average casein micelle size [21]. Furthermore, cheese prepared from milk containing the BB variant of κ-CN has higher fat recovery and yield than that made with milk containing the AA variant [21]. Thus, cows that produce milk containing the BB variant of κ-CN are economically important from a cheese-making perspective, owing to the micelle-size-related benefits of this protein type.

The prevalence of allele B in Israeli Holstein cattle as tested in the early 1990s was about 17%, similar to the Holstein population worldwide [22]. Today, the prevalence of allele B has increased in the Holstein breed worldwide and in Israel. Ikonen et al. [2] concluded that allele E is associated with poor coagulation compared to the other alleles, and in some cases, the milk is useless for cheese making.

Most studies have found a significant effect of the B allele on the amount of CN in the milk and, accordingly, the presence of this allele was found to result in 9.5–14% less whey protein. In a study of Jersey cows [23], the prevalence of alleles E, A, and B was 10, 30, and 70%, respectively, and Zepeda-Batista et al. [24] found incidences of 3, 55, and 42% for these respective alleles in the Fleckvieh breed. Another study conducted in Czech Fleckvieh cattle found 5, 72, and 23% frequencies of the E, A, and B alleles, respectively [25]. They also found that cows with the AA genotype produce the highest quantity of milk among the genotypes. Cows with the BB genotype yielded the highest amount of milk protein relative to other genotypes. Cows with genotypes BB and BE yielded milk with the highest protein percentage. In Lithuania [26], frequencies of the E, A, and B alleles were 6, 80, and 14%, respectively, in Black and White Holsteins, and 18, 70, and 11%, respectively, in Red and White Holsteins. In addition, this study clearly showed shorter coagulation times and firmer cheese with milk from BB cows compared to AA and AB cows’ milk, while AE cows’ milk was clearly inferior to all other genotypes in both parameters. Coagulation quality can be improved by increasing the prevalence of the B variant of κ-CN and collecting direct data, such as rennet coagulation time (RCT), or performing genetic tests to genotype the cows. However, the latter approach is rarely performed. A quicker and more efficient way of estimating the prevalence of the B allele in advanced commercial dairy populations is to use information from the cow’s pedigree, especially sires, since only a very low number of sires are generally used each year. Today, AI associations worldwide publish the bulls’ genotype for κ-CN, which accounts for 11% of CN, so the farmer can also choose the inseminating bull according to its κ-CN genotype.

However, most studies have neglected to look at the effect of the different genotypes, and especially allele E, on milk coagulation parameters (MCPs). Our study hypothesis was therefore that cows with the B allele will show superior MCPs, whereas cows with E allele will have inferior MCPs. To test this hypothesis, our objectives were to analyze milk coagulation properties as a function of the cow’s κ-CN genotype, including time in minutes until the beginning of coagulation (RCT) and curd firmness after 60 min in volts as measured via an optigraph device and observed by a laboratory technician rated on a scale of 0–4.

## 2. Materials and Methods

We analyzed two datasets. Dataset 1 was used to estimate the κ-CN allele frequency in Holstein cow populations in Israel, based chiefly on the genotypes of the sires. A total of 1447 bulls and 4430 cows were genotyped between 2011 and 2021. We routinely use the Bovine 150K chip for genotyping bulls and cows, and one of the outputs is the κ-CN genotype. All genotyping was performed by Neogen (Lansing, MI, https://www.neogen.com/about, accessed on 1 January 2023) and BeadChips v3 (Illumina Inc., San Diego, CA, USA, https://www.illumina.com/science/technology.html, accessed on 1 January 2023). Dataset 2 was used to analyze MCPs in a group of cows (*n* = 359). Cows were selected from the herdbook according to their sire’s and grandsire’s allele for κ-CN to reach a balanced sample that included all genotypes. We selected 391 cows with sire genotypes AE, BE, and BB to have a sufficient number of cows presenting the different genotypes for the study. The selected cows were tested for their κ-CN genotypes from a hair sample (Neogen). Cows with a known κ-CN genotype were sampled according to the following protocol: Cows in mid-lactation (average of 148 d) and cows with an SCS (somatic cell score) lower than 4 (average of 2.03) were checked for clinical or subclinical mastitis using the California mastitis test (CMT) on the quarter level. If a quarter showed a positive CMT result (1 or higher), we did not take a milk sample from this specific quarter and the quarter was removed from the MCP analysis. So, when taking samples, we used the quarter as the experimental unit. After we collected the milk sample, the experimental unit was the cow. In total, we tested 359 cows from 15 dairy farms.

A milk sample was collected (30–45 mL of a mixture of whole udder yield) and divided into 2 different samples for analysis as follows: The first sample was tested for SCC with a Fossomatic 360 (Foss Electric, Hillerød, Denmark) and gross milk composition, i.e., protein, fat, and lactose contents, with a MilkoScan FT6000 (Foss Electric). These analyses were performed at the Israel Cattle Breeders’ Association laboratory (Caesarea, Israel). The second sample was tested for curd firmness after 60 min (CF-60) and RCT with an optigraph (Ysebaert, Frepillon, France). The curd firmness was observed by the same laboratory technician after every cheese-making session, who scored the curd from 0 (weak and more liquid) to 4 (hard and more stable). Samples (10 mL) were placed in wells and equilibrated at 30 °C. The coagulating enzyme was Fromase 15 TL (0.5 mL, Gist-Brocades NV, Delft, The Netherlands), diluted (1:100) to achieve clotting within about 900 s in bovine milk [27].

### Data Analysis

Dataset 2 included 359 cows. The MCPs were analyzed by the GLM procedure of SAS (2009, SAS Institute Inc., Cary, NC, USA). The analysis model was:

Yijklm = Hj + Lk + Gl + SCSijkl + Mijkl + PFijkl + PPijkl + Dijkl + eijklm,

where the dependent variables, Yijklm, are the CF-60 and RCT of cow i in herd j of parity k with genotype G; Hj is the herd effect j; Lk is the parity effect k (1,2,3,4+); Gl is the genotype effect l (AA, AB, AE, BB, BE); SCSijkl, Mijkl, PFijkl, and PPijkl are the effects of test day SCSs, milk, % fat, and % protein records of cow i; D is the days in milk effect (DIM) on the date of the milk sample; and eijklm is the random residual.

Multiple comparisons for significance among the genotype effects were conducted via the Bonferroni procedure. All first-degree interactions were tested and found to be non-significant and were therefore excluded from the final models. The results for a level of a specific variable included in the model were based on least square (LS) mean values, as presented by Lavon et al. [28].

## 3. Results

The frequencies of κ-CN alleles and genotypes from dataset 1 are presented in Table 1. The allele frequencies of A, B, and E were 57.1, 40.3, and 2.6%, respectively. The genotype frequencies of AA, AB, AE, BB, BE, and EE were 32.47, 46.33, 2.84, 16.05, 2.28, and 0.03%, respectively.

The distribution of alleles and genotypes in the cows from dataset 2 is summarized in Table 2. Our sampling protocol resulted in >40 cows for all genotypes, except for EE, which was very rare in the population. The allele frequencies of A, B, and E were 43.31, 44.43, and 12.26%, respectively. The genotype frequencies of AA, AB, AE, BB, BE, and EE were 17.83, 39.55, 11.42, 18.11, 13.09, and 0.00%, respectively.

Test-day results for milk yield, milk solids content, SCSs, and DIM for each genotype are summarized in Table 3. Cows with the AA genotype had lower percentages of milk fat and protein, but differences between the genotypes for the test-day parameters were not significant.

**Figure 1:**
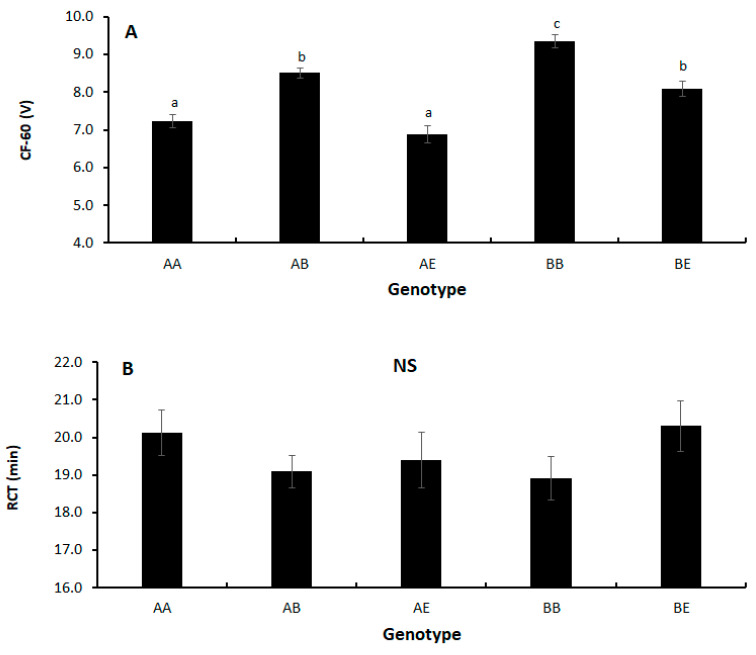
Genotype effect on (**A**) curd firmness after 60 min (CF-60) and (**B**) rennet coagulation time (RCT). Values are LS means ± SEM. Different letters indicate significant difference (*p* < 0.05).

**Figure 2:**
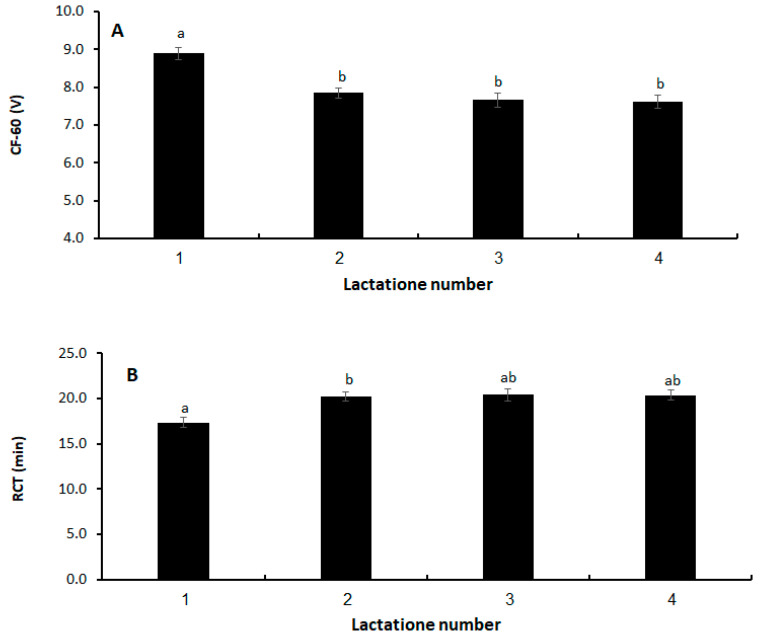
Effect of lactation number on (**A**) curd firmness after 60 min (CF-60) and (**B**) rennet coagulation time (RCT). Values are LS means ± SEM. Different letters indicate significant difference (*p* < 0.05).

Milk coagulation traits (CF-60 and RCT) were affected by the κ-CN genotype (Figure 1A,B) and by lactation number (Figure 2A,B). Cows with genotype BB had the highest curd firmness, followed by cows with the B allele in combination with A or E (*p* < 0.05; Figure 1A). Cows with the AA or AE genotype (without the B allele) had the lowest curd firmness (*p* < 0.05; Figure 1A). Milk from cows with the B allele took less time to coagulate (lower RCT), especially for BB cows, but the difference was not significant (Figure 1B). A higher curd firmness was obtained from primiparous cows’ milk compared to that of multiparous cows, with no difference found among the multiparous cows (*p* < 0.05; Figure 2A). The RCT was lower for the primiparous vs. multiparous cows, but the difference was only significant compared to second-lactation cows (*p* < 0.05; Figure 2B).

Table 4 presents the model effects and SE and *p*-values for the effects of genotype, lactation number, log SCC, milk yield, milk fat and protein percentages, and DIM on RCT and CF-60. With respect to the former, there was no difference among genotypes. In contrast, cows in first lactation showed a reduced time to coagulation than the older cows (Table 4; *p* < 0.0001). There was significantly higher curd firmness as determined by CF-60 for cows with the BB genotype. The order of curd firmness (high to low) was BB > AB > BE > AA > AE (Table 4; *p* < 0.0001). In addition, milk from first-lactation cows gave a higher curd firmness than that of older cows (Table 4; *p* < 0.0001).

The 10 cows with the highest and lowest curd firmness scores are given in Table 5. Of the 10 cows with the highest curd firmness, 9 had the B allele and 6 had the BB genotype (Table 5). Of the 10 cows with the lowest curd firmness, only 1 cow had allele B and all of the cows had the A or E allele. This clear distinction was not apparent with respect to the RCT values.

## 4. Discussion

This study presents the current distribution for κ-CN genotypes and alleles in the Israeli dairy population. Most publications show an advantage for the B allele in protein and CN content. The prevalence of allele B in Israeli Holstein cattle in the early 1990s was about 17% [22]. In the current study, the prevalence of the B allele has more than doubled to around 40% in the entire population. This strong increase stems from the advantage of the B allele for protein content, which is the major objective in the Israeli breeding index [29]. In contrast, the prevalence of the E allele has decreased from about 6% to 2.6%. Cheese making requires milk coagulation and development of syneresis. The dairy industry pays a great deal of attention to MCPs, principally because the amount of milk used for cheese production is growing worldwide (International Dairy Federation, 2020). In the last decade, the fraction of total milk destined for cheese production has increased by about 10% in the European Union and North America, and it is now slightly higher than 50% in the EU and slightly lower in North America. An increase in the amount of milk used to manufacture cheese has been reported in other European countries, Oceania, and Latin America, whereas a much lower amount is used as compared to Asia and Africa. The B variant of κ-CN is associated with a higher protein percentage compared to the E variant, with the A variant being intermediate between the two [20]. Milk production is correlated with κ-CN genotypes in the order AB > AA > BB [30]. The order of κ-CN genotypes as they relate to protein content is BB > AB > AA [31], or AB > AE > AA [32]. However, Lodes et al. [33] found the opposite order, i.e., AA > AE > AB. In addition, Ikonen et al. [2] reported that the EE, AE, and BE variants contribute to a high milk yield but a low protein percentage. The BB variant was found to be positively correlated with milk and milk protein production during the first lactation [34]. In the current study, we did not find any correlation between genotype and milk level or milk solids content. Comin et al. [35] reported that κ-CN is the most important milk protein in rennet coagulation, as it is the key to CN micelle stability, providing steric and electrostatic repulsion between micelles to prevent aggregation through the surface ‘hairy’ layer of micelles [36]. The B variant was found to be associated with a high milk quality in European cattle breeds [37] and, compared to the A variant, B is found to be associated with shorter RCTs [38]. Cheese formed using milk with the BB variant has a higher yield, protein content, and quality compared to the AB variant [22]. The different genetic milk protein variants and CN haplotypes have a major effect on the protein composition of milk. In general, the A allele of κ-CN is associated with a longer RCT and weaker curd [39]. We found a higher curd firmness in cows with the B allele as compared to those without it. Genotype BB had the highest curd firmness (CF-60), followed by AB, BE, AE, and AA with the lowest curd firmness. The RCT was lower, in agreement with higher curd strength, in cows with allele B, but this difference was not significant. Cow age (primiparous vs. multiparous) had a significant effect on the MCPs, where first-parity cows had a higher curd firmness and a lower RCT compared to older cows. Determination of MCPs in the entire cow population is not practical, due to the large effort and expense required. An alternative way of improving MCPs indirectly might be to favor the B allele of κ-CN in the selection of bulls for general service.

## 5. Conclusions

This study shows the result of the Israeli breeding program, which favors bulls and cows with higher percentages of milk solids. We noted a steep rise in the frequency of the B allele in the population, which can improve coagulation properties. The current study analyzed a large number of dairy cows with known genotypes for κ-CN. We found that the presence of the B allele for κ-CN is associated with superior coagulation parameters (CF-60 and RCT) compared to cows without the B allele. Cows with the AA or AE genotype had a lower curd firmness compared to cows with the BB, AB, or BE genotype. We also found that primiparous cows present superior coagulation parameters (CF-60 and RCT) compared to cows in their second, third, or fourth lactation. Inclusion of the κ-CN genotype in the Israeli selection index could further raise the frequency of the B allele in the population as a direct effect and have an indirect effect on the percentage of milk solids, leading to a faster rise in the B allele and improved MCPs for the milk industry in Israel.

## Figures and Tables

**Table 1:** Genotype frequency and κ-CN alleles from dataset 1.

Frequency (%)	n	Allele	Frequency (%)	n	Genotype ^1^
57.11	6706	A	32.47	1908	AA
40.28	4743	B	46.33	2723	AB
2.61	305	E	2.84	167	AE
			16.05	943	BB
			2.28	134	BE
			0.03	2	EE
100	11,754		100	5877	Total

^1^ A total of 5877 bulls and cows were genotyped using the Bovine 150K chip between 2011 and 2021.

**Table 2:** Genotype frequency and κ-CN alleles found in cows included in dataset 2.

Frequency (%)	N	Allele	Frequency (%)	n ^2^	Genotype ^1^
43.31	311	A	17.83	64	AA
44.43	319	B	39.55	142	AB
12.26	88	E	11.42	41	AE
			18.11	65	BB
			13.09	47	BE
			0	0	EE
100	718		100	359	Total

^1^ Cow genotype was tested in a hair sample taken from each cow. ^2^ Data of 359 cows from 15 different dairy farms.

**Table 3:** Test-day results for milk yield, milk solids contents, SCSs, and DIM according to cow genotype for κ-CN.

Genotype ^1^	n ^2^	Milk (kg)	Fat ^3^ (%)	Protein (%)	SCS	DIM
AA	64	42.6	3.18	3.20	1.8	135
AB	142	40.3	3.54	3.30	2.1	147
AE	41	39.7	3.41	3.30	2.2	161
BE	47	39.6	3.49	3.36	2.4	172
BB	65	43.5	3.43	3.27	1.8	140
Total	359	41.4	3.43	3.28	2.0	149

^1^ Cow genotype was tested in a hair sample taken from each cow. ^2^ Data of 359 cows from 15 different dairy farms. ^3^ Milk solids and SCCs were determined at the Israel Cattle Breeders Association laboratory.

**Table 4:** Effects of genotype, lactation number, SCS, milk yield, milk fat and protein percentages, and DIM on rennet coagulation time (RCT) and curd firmness at 60 min (CF-60).

Factor	Level	RCT	SE	Pr > |t|	CF-60 (V)	SE	Pr > |t|
Genotype				0.3409			<0.0001
	AA	1.36	0.848		−2.14	0.253	
	AB	−0.01	0.711		−0.85	0.212	
	AE	0.24	0.959		−2.44	0.286	
	BE	0.94	0.908		−1.27	0.271	
	BB	0.00	-		0.00	-	
Lactation number				0.0006			<0.0001
	1	−2.73	0.829		1.20	0.247	
	2	0.23	0.720		0.21	0.214	
	3	−0.02	0.810		0.05	0.241	
	4	0.00	-		0.00	-	
SCS		0.36	0.168	0.0349	−0.03	0.050	
Milk (kg)		−0.11	0.040	0.0064	0.05	0.012	<0.0001
Fat (%)		−1.03	0.413	0.0132	0.77	0.123	<0.0001
Protein (%)		1.67	0.979	NS	2.69	0.292	<0.0001
DIM		0.01	0.004	0.0006	0.0028	0.001	0.0237

**Table 5:** Effect of κ-CN genotype on milk coagulation parameters.

10 Cows with the Highest CF-60 Scores				10 Cows with the Lowest CF-60 Scores			
Visual Index	RCT	CF-60	Genotype	Visual Index	RCT	CF-60	Genotype
3.7	15.8	14.6	BB	2.8	14.3	4.2	AE
3.7	19.5	14.4	BE	1.0	21.9	4.2	AA
3.7	18.5	14.1	AA	2.5	19.1	4.1	AE
3.3	16.1	13.4	AB	3.4	17.3	4.0	AE
3.8	22.8	13.2	BB	2.6	18.6	3.8	AE
3.8	16.4	13.0	BB	0.0	42.4	3.4	AB
3.7	21.1	12.8	AB	0.0	42.1	3.4	AE
3.9	14.9	12.8	BB	3.5	12.7	3.3	AE
3.6	20.8	12.3	BB	1.9	13.7	2.8	AA
3.6	18.9	12.3	BB	0.2	31.6	0.3	AA

## Data Availability

Data are contained within the article.
